# Patterns of weight cycling in youth Olympic combat sports: a systematic review

**DOI:** 10.1186/s40337-022-00595-w

**Published:** 2022-05-25

**Authors:** Nemanja Lakicevic, Joseph J. Matthews, Guilherme G. Artioli, Antonio Paoli, Roberto Roklicer, Tatjana Trivic, Antonino Bianco, Patrik Drid

**Affiliations:** 1 Sport and Exercise Sciences Research Unit, University of Palermo, 90133 Palermo, Italy; 2 Research Centre for Life and Sport Sciences (CLaSS), School of Health and Life Sciences, Department of Sport and Exercise, Birmingham City University, Birmingham, UK; 3 Department of Life Sciences, Manchester Metropolitan University, Manchester, UK; 4 Department of Biomedical Sciences, University of Padova, 35122 Padua, Italy; 5 Faculty of Sport and Physical Education, University of Novi Sad, 21000 Novi Sad, Serbia

**Keywords:** Rapid weight loss, Rapid weight gain, Children, Adolescents, Health, Performance

## Abstract

**Background:**

Patterns of weight cycling in adult combat sports have been extensively studied, yet data on this matter in youth combat athletes is rather scarce.

**Methods:**

PubMed, EBSCOhost and Web of Science were used to retrieve relevant data. Eligible studies had to record the methods used to elicit rapid weight loss (RWL) and/or record the oscillations in bodyweight during the RWL phase. Only studies conducted in the context of an official competition were considered for inclusion in the present review.

**Results:**

RWL is highly prevalent in children and adolescent combat athletes, ranging from 25 to 94% depending on the type of combat sport, age and level of competition. These athletes regularly prompt RWL by increasing exercise frequency and intensity, decreasing fluid and food intake, training in impermeable suits and using sauna frequently. Overall, the magnitude of RWL was ranging from ~ 1% to 6.3 ± 3.7% with significant RWL variations within individual studies and individuals within those studies.

**Conclusion:**

Acquired data indicated that RWL patterns in young combat athletes are similar to those found in their adult counterparts. Knowing that childhood and adolescence are critical periods for growth and development, RWL needs to be stringently regulated and ideally banned in this population.

## Summary

Based on the data obtained through systematic search of the literature, it appears that the majority of young combat sports athletes engage in alarming weight cutting behaviors that can have serious ramifications both short- and long-term and even cause death as seen in American collegiate wrestlers in the past. Accordingly, until further research answers pertinent questions and revised guidelines for youths are published, parents, practitioners, coaches and adult athletes should take a conservative approach when discussing potential weight management strategies with young combat sports athletes whenever possible, as normal growth and development of these young athletes should be the utmost priority.

## Introduction

Weight categories in combat sports aim to give competitors a fair chance by matching opponents with similar anthropometric characteristics and physical abilities [1]. To achieve the desired body weight for competition, many combat athletes engage in weight cycling, involving periods of rapid weight loss (RWL) and rapid weight gain (RWG), typically referred to as “making weight” or “yo-yo” dieting. This practice has led to several fatalities [2] and raised concerns in both the scientific community and lay public [3]. A robust body of evidence shows that RWL is widespread in combat sports but can vary in prevalence and the weight loss methods used [4]. A striking finding is the magnitude of body mass that some athletes reduce in the final days before competition. A recent survey of British mixed martial arts examined athletes from various weight classes and showed that these athletes lost 9 ± 2% of body mass in the week before competition and a further 5 ± 2% in the final 24 h before weigh-in [5]. Equally striking is the magnitude of RWG in youth combat sports, where some junior wrestlers were able to gain 16.73 kg in under 7 h [6]. The short-term health effects of such weight loss and dehydration have been well documented (for a review, see Burke et al. 2021) [7]. The long-term effects, however, are less well studied, but there is some evidence that chronic weight cycling may lead to greater weight gain later in life [8], which could be mitigated if athletes maintain high levels of physical activity after retirement [9], unfortunately this is not always possible, and many athletes are physically inactive once their sporting career ends [10].

While RWL has been extensively studied in adult athletes, much less is known about the prevalence, methods, and risks of RWL in youth combat athletes. Of concern, is a case report from the United States that documented a five-year-old child making weight for a wrestling competition [11]; similarly, judokas in Israel and Brazil reported that they began cutting weight as young as four and nine years-old, respectively [12, 13]. As weight categories are often classified at the onset of adolescence, growth and maturation over the following years means that most adolescents compete in a category below their natural physiological body weight [15] to remain in the lowest weight category possible. Exposure of potentially harmful RWL behaviors to children and young athletes may have negative effects on bone health, metabolic health, menstrual function, and psychological health due to sustained periods of low energy availability [14]. During the growth phase, energy and nutrient restriction can lead to delayed prepubertal development, particularly in female athletes [15]. Another concern is the risk of disordered eating (DE) and eating disorders (EDs), as these often develop during adolescence, and athletes competing in weight-sensitive sports are already at an increased risk [16]. It is clear we need to develop a thorough understanding of RWL in youth combat athletes to help reduce acute and chronic health risks and avoid perpetuating the cycle of RWL that may continue into adulthood.

The aims of this paper, therefore, were to (1) Systematically review the literature on methods and magnitudes of RWL and RWG in youth Olympic combat sport athletes ((as these are far more popular than non-Olympic and represent 25% of medals earned at the Summer Olympics [1])) and (2) Propose strategies to reduce potentially harmful RWL behaviors. Based on the well established data on weight cycling in adult combat sports athletes, we hypothesized that this phenomenon will be highly prevalent in their younger counterparts.

## Materials and methods

To ensure transparent and complete reporting, this review followed the Preferred Reporting Items for Systematic reviews and Meta-Analysis 2020 guidelines [17].

### Eligibility criteria

Inclusion criteria were limited to athletes participating in Olympic combat sports (judo, wrestling, boxing, taekwondo, and karate), aged ≤ 19 years (children and adolescents as defined by the World Health Organization [18]), and studies had to report the methods used to elicit RWL and/or report changes in bodyweight (kg, lbs, or %) during the RWL or RWG phase. Literature defines RWL (in adult combat athletes) as a 5% weight loss achieved 5–7 days [19]. However, due to the scarcity of the RWL-related data being available in children and adolescents, we decided not to have a norm on the magnitude or duration of RWL. Studies which reported RWL data across multiple age ranges (e.g., including those > 19 years) were included if age-relevant data were reported separately and could be extracted for the present review. All studies were observational by nature and included longitudinal and cross-sectional designs. Controlled studies, such as those where the researchers impose a RWL target on the participants, were excluded. Only original studies written in English and published in peer-reviewed journals were considered for inclusion; other publication types such as reviews, meta-analyses, conference abstracts, and books, were excluded.

### Information sources, search strategy and selection process

A systematic electronic search of PubMed Central, EBSCOhost, and Web of Science was performed from the earliest record to 21 November 2021. Search strategies were developed using key text words related to the population and outcomes: ‘‘rapid weight loss OR rapid weight gain OR making weight OR weight cutting” – AND “judo OR wrestling OR boxing OR taekwondo OR karate”. Reference lists and citations of included studies were searched for additional eligible publications.

Retrieved articles were imported into EndNote (Clarivate Analytics, Jersey, UK, ver. 9.1.), and screened independently by two authors (NL & RR) using a three-step procedure: (1) titles reviewed, (2) abstracts reviewed, and (3) full-texts reviewed. Disagreements (n = 2) were resolved through consensus-based discussion between the two reviewers; remaining disagreements (n = 1) were referred to a third reviewer (PD). Reviewers were not blind to the study authors or institutions during the selection process.

### Data extraction and data synthesis

We extracted data using a standardized form based on the guidelines from the Centre for Reviews and Dissemination [20]. Data items included: (1) Sample size and participant characteristics (age, height, combat sport, competitive level, nationality); (2) Body mass (baseline, weigh-in, pre-competition); and (3) Methods of rapid weight loss (types, natural frequencies, percentage used etc.). For continuous outcomes (e.g., body mass) all studies used the same measurement scale (kilograms or pounds), and mean difference values were reported. Findings are synthesized as a narrative summary using tables of evidence. Study authors were not contacted for raw data or further information.

### Assessment of risk of bias in individual studies

The Newcastle–Ottawa Scale for assessment of quality of nonrandomized studies [21] was used to assess the risk of bias in individual studies. This scale has been validated previously and explores quality in three broad perspectives: study groups selection; the comparability of the groups; and the ascertainment of either the exposure or outcome of interest for case–control or cohort studies respectively.

## Results

### Study selection

**Fig. 1:**
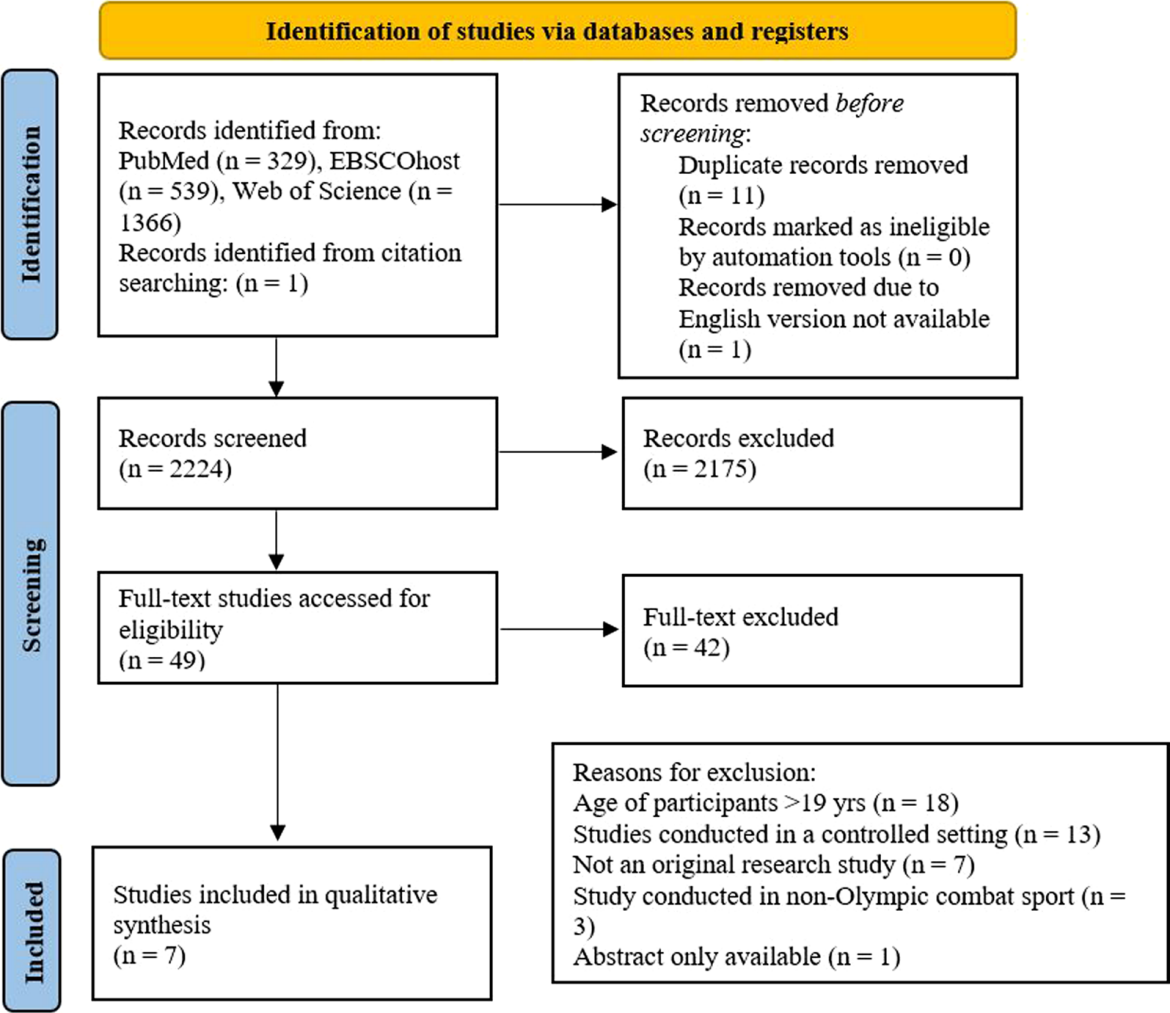
PRISMA flow-chart: search results

The search strategy and selection process resulted in 7 full-text studies [6, 13, 22–26] for inclusion in the review (Fig. 1).

### Study and participant characteristics

The included studies were published between 2004 and 2020 (Table 1). Data were available for three Olympic combat sports (wrestling n = 3, judo n = 3, boxing n = 1) which included data from 3135 participants (28 females, 469 males; study with the largest sample (n = 2638) did not report on the sex characteristics of the participants). There were no studies on the prevalence and methods of RWL or RWG in adolescents in karate and taekwondo. Five studies recorded RWL, two studies recorded RWG, no studies recorded both outcomes; and five studies reported methods used to elicit RWL. All studies were conducted with participants preparing habitually for a competition.

**Table 1:** Sample characteristics and weight cutting protocol

Reference	Sample size	Age (years)	Height (cm)	Pre-RWL/RWG body mass (kg)	RWL or RWG	Methods of RWL	Magnitude of RWL/RWG (kg,%)	RWL/RWG duration
Alderman et al. [6] (US-46 states)	N = 2638 45 wrestlers completed weight management questionnaire	15–18	NA	NA	RWG	Increased exercise, sauna use, training in impermeable clothing, use of laxatives	3.4 ± 1.8 kg (4.1%) [− 2.68 to 16.73 kg (− 2.1% to 13.4%)]	3–7 h
Berkovich et al. [13] (Israel)	N = 108 judokas (males)	14.6 ± 1.6 (range 11.1–17.5)	168.8 ± 10.2	58 ± 12.1	RWL	Increased exercise, heated room training, training in impermeable clothing, sauna use, fluid restriction, food restriction, gradual dieting, fasting	1.5 ± 1.1 kg (no percentage available)	8.0 ± 5.4 days
Boisseau et al. [26] (Belgium)	N = 9 judokas (females)	16.1 ± 0.3	163 ± 2	58.9 ± 3.6	RWL	Fluid restriction, training in impermeable clothing	2%	7 days
Karnincic et al. [22] (Croatia)	N = 77 wrestlers (males)	16.0 ± 0.8	Lightweight = 167.8 ± 7.2 Middleweight = 176.3 ± 6.8 Heavyweight = 179.2 ± 3.5	Lightweight = 55.2 ± 13.6 Middleweight = 65.5 ± 2.8 Heavyweight = 81.8 ± 8.7	RWL	NA	1.25%	7 days
Nascimento et al. [25] (Brazil)	N = 186 wrestlers (males)	13.31 ± 0.6	53.64 ± 0.6	NA	RWL	Fluid restriction, training in impermeable clothing, increased exercise, food restriction	1 kg (~ 1.8%)	7 days
Viveiros et al. [24] (Brazil)	N = 31 Wrestlers (males 15; females 19)	13 ± 2	NA	NA	RWG	Increased exercise, food restriction	females = 6.3 ± 3.7%; males = 3.1 ± 1.8%	24 h
Zubac et al. [23] (Europe)	N = 83 boxers (males)	17.1 ± 0.9	176 ± 8 cm	68.5 ± 12.9	RWL	Food restriction, training in impermeable clothes, sauna use	2–5%	< 7 days

*N* number of participants; *NA* not available; *RWL* rapid weight loss; *RWG* rapid weight gain

### Risk of bias in individual studies

Quality assessment of included studies showed overall fair quality of the included studies (Table 2). The key issue was the absence of a control group in each study, which is expected as all studies were conducted in a real-world setting and not during a laboratory or controlled experiment.

**Table 2:** Risk of bias

	Selection				Comparability^1^	Outcome			
Study	A	B	C	D	E	F	G	H	Total (9/9)
Alderman et al. [6]	*	0	0	*	0	0	*	*	4
Berkovich et al. [13]	*	0	*	0	*	*	*	*	6
Boisseau et al. [26]	*	0	0	*	**	0	*	*	6
Karnincic et al. [22]	*	0	*	*	*	*	*	*	7
Nascimento et al. [25]	*	0	*	*	*	*	*	*	7
Viveiros et al. [24]	*	0	*	*	*	*	*	*	7
Zubac et al. [23]	*	0	*	*	**	*	*	*	8

*A* representativeness of the exposed cohort, *B* selection of the non-exposed cohort, *C* ascertainment of the exposure, *D* demonstration that outcome of interest was not present at start of study, *E* comparability of cohorts on the basis of the design or analysis, *F* assessment of outcome, *G* was follow-up long enough for outcomes to occur, *H* adequacy of follow up of cohorts

*A single point allocated to a particular study

^1^A maximum of two stars (points) can be given for Comparability

### Prevalence of RWL

Prevalence of RWL was high across combat sports: 42 to 77% of wrestlers [22, 24, 25] and 80% of judokas [13]. One exception was in boxers, where the prevalence of RWL was only 25% [23]. Two studies did not report on the prevalence of RWL [6, 26].

### Magnitude of RWL/RWG

Two studies reported RWG [6, 24] with both studies directly measuring body mass; five studies reported RWL [13, 22, 23, 25, 26] all of which were based upon self-report data from questionnaires. The RWL duration ranged from 7 to 8.0 ± 5.4 days [13, 22, 23, 25, 26]; and the duration permitted for RWG ranged from 3 to 7 h [6, 24]. For RWG, 3.4 kg (4.1%) weight gain in 3–7 h was reported in US wrestlers [6], compared with 6.3 ± 3.7% and 3.1 ± 1.8% in under 24 h in female and male Brazilian wrestlers, respectively [24].

Reported RWL showed comparably smaller amounts of body mass change. Across a 7-day RWL period, Croatian wrestlers reduced their body mass by 1.25% [23], Brazilian wrestlers by 1.8% [27], and French judokas by 2%, whereas Israeli judokas reduced their body mass by 1.5 kg in 8.0 ± 5.4 days [29]. In boxing, participants coming from twelve European countries engaged in 2–5% weight loss undertaken in less than seven days [23].

### Methods of RWL

Methods of RWL were similar across all studies, despite the differences in sport and geographical regions. In Brazilian wrestlers, increased exercise and food restriction were the most common methods of RWL [24, 25], while American wrestlers favored increased exercise (running, cycling, swimming) along with frequent use of sauna, training in impermeable clothing, and taking laxatives [6]. Notably, the study with the largest sample size (*n* = 2638) only collected data on RWL methods from a subset of wrestlers (*n* = 45) [6]. No data on RWL methods were available for Croatian wrestlers [22].

In French [26] and Israeli judokas [13], food and fluid restriction, along with training in impermeable clothing, were the most dominant methods used to induce RWL; increased exercise, training in heated rooms, and sauna use were reported only by Israeli judokas. Similar trends were observed in European boxers where food restriction, training in impermeable clothes, and sauna use were commonly used, as well as weight loss supplements [23].

## Discussion

This systematic review investigated the methods, magnitude, and prevalence of RWL and RWG in youth Olympic combat athletes. Based on the results from seven studies, we found that RWL and RWG behaviors in youth Olympic combat sport athletes are similar to those in adult combat sport athletes [27], albeit with a smaller overall magnitude of body mass manipulation (~ 1% to 6.3 ± 3.7% in children and adolescents vs. 5–10% or even more commonly found in adults). Of concern, is the evidence that youth combat sport athletes use potentially harmful RWL methods, such as training in impermeable clothing, fluid restriction, sauna use, and laxative use [6, 13, 23–25]. Some of these methods have been implicated in previous fatalities and hospitalizations in adult combat sport athletes [2], which highlights a potential health, safety, and welfare issue.

Unsurprisingly, multiple studies included in this review have reported on adverse symptoms associated with RWL [6, 22, 23]. Alderman and colleagues [6] found that wrestlers engaging in RWL experienced headache, dizziness, nausea, hot flashes, nose bleeds and to a smaller degree feverish, disorientation and increased heart rate. In a study by Zubac, Karnincic and Sekulic [23], 30% of boxers experienced fatigue and 21% experienced myalgia during RWL. Contrary, Karnincic, Baic and Slacanac [22] focused on psychological effects of RWL and found significant increases in depression, anger and fatigue among young wresters partaking in RWL.

Overall, the magnitude of RWL was ranging from ~ 1% to 6.3 ± 3.7% with significant RWL variations within individual studies and individuals within those studies. It can be implicated that the greater the magnitude of RWL, the greater the risk of health and performance consequences since adequate caloric and micronutrient intakes are critical for developing children and adolescent athletes [28]. Intentional energy deficit and dehydration during physical training and competition in childhood or during adolescence could disturb metabolic and hormonal regulations affecting growth, maturation, body composition, menstrual cycle and reproductive capacity, and may increase the risk of injuries such as stress fractures [29]. Within participants included in this review, two adolescent girls were amenorrheic for a year or longer even before undergoing RWL [26].

Prevalence of RWL ranged from 25 to 80% within included studies, with higher prevalence in older athletes (94%) [13]. Low prevalence of RWL methods (25%) identified in young boxers is likely due to the body-mass span in boxing being relatively narrow while light weight classes are closely aligned (ten different weight-classes) [23]. The current rules and regulations in boxing whereby a wide spectrum of weight classes is offered to the competitors might be a pathway that facilitates gradual rather than RWL as seen in Olympic-style boxing. However, height might be related more to competitive success than weight as limb-length is crucial in striking combat sports and therefore recent studies indicated that weight making should be replaced with height categories in some combat sports [30, 31]. In addition, RWG is noted to likely be less beneficial in striking combat sports, where competitive success depends more on tactical executions of movement, footwork, speed, and successfully landing blows to an opponent’s body [23, 32] while it is linked to competitive success in grappling sports where the goal is to manipulate an opponent’s body and impose one’s own body weight [33]. It is of great significance to determine whether RWL provides real or perceived performance benefits in combat sports as this is likely to underpin motives of athletes to undergo RWL [7].

Within included studies, only one study reported dietary intake during RWL [26]. Data showed that ~ 20% caloric deficit was applied in order to induce RWL and caloric intake was not sufficient to support total energy expenditure. This deficiency was mainly attributed to significantly reduced carbohydrate intake when compared to three weeks prior to RWL week. However, water intake was also reduced by 8.5% which led to lower urinary excretion (-58%) during RWL week. The participants had lower daily intake of calcium, magnesium, copper, iodine, vitamin E, and fiber when compared to recommended values for adolescents of similar age. Protein intake was higher in RWL week when compared to two weeks prior coming dominantly from animal sources. The same study found that two out of nine girls were amenorrheic for 1 year even before engaging in RWL. These findings are largely in alignment with data found by Lingor and Olson [30] where wrestlers of similar age halved their fluid intake one day prior to competition compared to other days during the week with two individuals fasting for more than 24 h and 1 individual reported having 200 ml of fluid per day to make weight during the weeks when diet was recorded, which can lead to acute kidney damage [31]. After intentional food and fluid deprivation, not surprisingly, athletes in this study were also deficient in calcium, magnesium, iron, zinc, copper, iodine, and vitamin E.

It is possible that a focus on RWL from a young age, with the use of potentially harmful methods, contributes to the development of DE and EDs in youth combat athletes. In a large group of interscholastic wrestlers, Oppliger et al. [31] reported that 1.7% of athletes answered questions consistent with all five criteria for bulimia nervosa—a rate higher than expected for adolescent males. In addition, nearly half of the sample exhibited RWL practices similar to those who met the ED criteria. Likewise, another study reported that among the National Collegiate Athletic Association male athletes, 7% of wrestlers had manifested ED behaviors [34]. Moreover, a study conducted in Germany investigated eating behaviors in wrestlers in the lower weight categories and found that 52% had a history of bingeing and 11% of the sample fit the Eating Disorder Inventory profile for a subclinical ED [35]. The authors also suggested that light-weight male wrestlers can be considered as high-risk athletes for developing ED. Similar findings were observed in a 14-year-old boxer who engaged in severe weight cutting behaviors and was diagnosed with bulimia nervosa [36]. Of particular concern, our review has shown that RWL has been mainly encouraged by coaches, with very few athletes seeking advice from health professionals such as physicians or nutritionists as shown within included studies [13, 25]. This environment of influence mirrors the case in adult combat sport populations [37, 38]. In the absence of evidenced based education programs or professional intervention, it is evident RWL practices are routinely passed from coach to athlete, and from athlete to athlete, with such practices centered on tradition rather than scientifically-orientated methods [39].

A growing body of evidence is emerging to indicate complications of future cardiometabolic health in those who were normal weight prior to weight cycling compared to those who were obese [23]. Within the present review, three studies showed that their participants had normal body mass index values prior to engaging in RWL [6, 13, 26]. A particularly troubling matter was found in Israeli judokas where some of them were making efforts not to gain weight to remain in a lower weight class category for periods of 2 years or longer [13]. Though, it is possible that several athletes had completed puberty and linear growth, and had achieved their final adult weight, but the majority of them reported using RWL to remain in their weight category. These growing adolescents who intentionally diet to prevent weight gain to compete in the lowest weight class possible can impair natural growth rates and should be discouraged from this practice whenever possible. Paradoxically, despite regular dieting in high-school wrestlers scientists detected an average 6.2 kg weight gain after each season [40].

### Strengths and limitations

To our knowledge, this is the first systematic review that looked at weight cycling patterns in child and adolescent combat athletes. We thoroughly searched the existing literature and set stringent eligibility criteria to identify the best quality evidence available. Our study examined 3135 combat athletes coming from nearly 20 different countries spanning across 4 different continents. Similar “weight cutting” patterns identified allowed us to derive conclusions among overall sample, despite vast cultural and societal differences posed by their place of birth.

Contrary, due to the scarcity of the available evidence we included studies that had various degrees of weight loss that was conducted over a varied period of time. Therefore, even though inter study comparisons were made in the results section, those are subject to criticism as significant time differences allocated to RWL or RWG were found within included studies. Moreover, baseline pre-RWL body mass was not available for studies where athletes took part in RWG. Also, some studies did not report on sex and height of athletes involved in the study. It is important to emphasize that our search yielded only 7 studies, illustrating that the topic of weight cycling in youth combat sports is in its infancy and thus more research in this field is needed. Even though we analysed data from more than 3000 participants, we only included studies published in English, so there is a possibility we have omitted some studies published in other languages.

### Health over performance

It is reasonable for athletes to utilize all legal tools to excel their performance and gain advantage over their opponents, but in the case of RWL/RWG we believe that athletes, parents, coaches and senior athletes should take a conservative approach when discussing weight cutting in the context of young combat athletes whenever possible [41]. The available evidence on RWL in children and adolescents is just starting to emerge and further research that answers pertinent questions and revised guidelines specifically for youth combat sport athletes are needed. Therefore, until more evidence is available, we recommend refraining from interventions aiming to promote weight cycling in young combat sports athletes and focusing on mastering sport-specific skills necessary to excel in a given combat sport. If adequate nutrition to ensure proper development of these young athletes is provided, it will enable them to reach their full potential both physically and mentally [28]. Combat athletes who are competing at an appropriate body composition achieved with scientifically based training and nutrition will maximize their performance without needing to engage in pre-competition RWL [19].

While individual efforts precluding RWL are important, competition regulations are key and have been shown to be successful in collegiate wrestlers in the United States whereby less seasonal variation in body weight, better retention of fat free mass, and a substantial reduction in RWG between the weigh-in and competition were noted when compared seasons prior implementation of these rules [7]. This is well illustrated in the case of young American wrestlers who return to prohibited weight cycling methods once they compete on an international level [6]. It is for this reason that experts in the field of combat sports science are calling for implementation of weight loss regulations similar to the National Collegiate Athletic Association wrestling in other combat sports [42].

### Practical recommendations and conclusions

The recent American College of Sports Medicine Expert Consensus Statement on weight loss in weight-category sports gave recommendations for safer weight making practices [7]. These included discouraging of weight making among minors and the youngest of participants, which we support, but there is a need for guidance specific to youth combat athletes that reduces the focus on bodyweight. We therefore propose the following:

- Coach, athlete, and parent education should be prioritized; with education materials made available by sport governing bodies as part of coaching qualifications and athlete registration
- For younger athletes (≤ 13 years, outside the Youth Olympic games age range), there should be less focus on weight-categories to standardize competition; instead, opponents of a similar size can be matched depending on their bodyweight on arrival (applicable for some tournament formats)
- Striking sports may consider the use of height categories, particularly where limb-length discrepancies may lead to greater inequality between competitors than bodyweight (e.g., taekwondo or boxing)
- Prohibiting dehydration-based RWL methods and athletes engaging in these to be prohibited from competition

## Conclusion

In summary, we found that RWL and RWG behaviors in youth Olympic combat sport athletes are similar to those in adult combat sport athletes, albeit with a smaller overall magnitude of body mass manipulation. RWL includes potentially harmful methods, some of which have led to previous fatalities and hospitalizations in adult combat sport athletes. We therefore need education and rule-based preventative strategies to avoid perpetuating the cycle of RWL from adolescence into adulthood. The current RWL landscape in youth Olympic combat sport athletes highlights a potential health, safety, and welfare issue, which warrants further investigation.

## Data Availability

Not applicable.

## Abbreviations

RWL: Rapid weight loss
RWG: Rapid weight gain
